# A citizen science model turns anecdotes into evidence by revealing similar characteristics among Gifted Word Learner dogs

**DOI:** 10.1038/s41598-023-47864-5

**Published:** 2023-12-14

**Authors:** Shany Dror, Ádám Miklósi, Andrea Sommese, Claudia Fugazza

**Affiliations:** 1 Department of Ethology, Eötvös Loránd University, Pázmány P. s 1c, 6th Floor, Budapest, 1117 Hungary; 2 Doctoral School of Biology, Institute of Biology, ELTE Eötvös Loránd University, Budapest, Hungary; 3 MTA-ELTE Comparative Ethology Research Group, Budapest, Hungary; 4 ELTE-ELKH NAP Comparative Ethology Research, Budapest, Hungary

**Keywords:** Animal behaviour, Zoology

## Abstract

Dogs that have a vocabulary of object labels (Gifted Word Learner dogs—GWL dogs) have great potential as a comparative model for studying a variety of cognitive mechanisms. However, only a handful of studies, with a small sample size of 1 or 2 dogs, have examined this phenomenon. GWL dogs appear to share many of the same distinctive characteristics, but due to their rarity, it is not clear if these similarities are only anecdotal or indeed reflect characteristics that are similar in these rare individuals. Here we present the first study conducted on a relatively large sample of 41 GWL dogs that were recruited and tested using a citizen science model. After testing the dogs' receptive vocabulary of toy names, we asked the owners to complete a questionnaire about their and their dog’s life experiences. Our findings highlight several characteristics that are shared among most GWL dogs, such as their learning speed, their large vocabulary, and that they learned the names of the toys spontaneously, without the explicit intent of their owners. Our findings validate previous anecdotal evidence on common characteristics of GWL dogs and supply additional support to the hypothesis that these dogs represent a unique group of dogs.

## Introduction

Dogs are a popular model for studying social cognition^1^ as a result of their evolutionary and developmental history in human society^2^. Dogs possessing a receptive vocabulary of object labels, that is, the ability to associate a verbal label with an object, have the potential to serve as a comparative model for investigating various cognitive mechanisms. These may include the acquisition of object labels (e.g.,^3,4^), the understanding of labels as referential communicative signals by non-human species (e.g.,^5,6^), and the perception of labels as category representatives^7,8^. In addition, the dogs’ knowledge of object labels can be utilized to examine whether they have an attentional bias towards certain features of objects^9^. The influence of verbal labels (and, ultimately, language) on mental representations can be examined by comparing dogs with knowledge of object labels to naïve dogs^10^.

However, despite the popularity of dogs as companion animals, and in contrast to their increasing popularity as comparative models for social cognition, only very few studies have been published on dogs’ knowledge of object labels. Most of these studies were conducted on a very small sample size of 1 or 2 dogs (e.g.,^4,7,11^) because, contrary to owners’ reports, it appears that most of the typical companion dogs do not demonstrate behavioural evidence of knowing object labels at the individual level^12^. The majority of the attempts to specifically train dogs to learn object labels also failed^13,14^ (but see^7,15^), suggesting that this ability is present in only a small group of uniquely Gifted Word Learner (GWL) dogs^13^.

A look into peer-reviewed papers on GWL dogs reveals that, although the studies included only a few individual dogs, these seem to share many characteristics. Overall, not counting our publications, eight studies were published on the topic. These included only 6 dogs that were often repeatedly observed in several cognitive tests. The 6 GWL dogs included 5 Border collies, of which 3 males (*Rico*^4,6,11^, *Paddy*^5,6^, and *Gable*^9^) and 2 females (*Betsy*^5,6,11^, *Chaser*^7,16^) and one female Yorkshire Terrier (*Bailey*^17^). All 6 dogs knew the names of over 40 dog toys when the researchers tested them. This is in contrast with dogs that were reported to know the names of only 3–5 objects, mostly of utilitarian use (such as a spoon, a brush, or a key)^15,18^. In addition, all but one of the GWL dogs (*Chaser*^7,16^) were not intentionally trained by professionals to learn the names of objects to participate in an experiment. Instead, they learned the names of the toys during play interactions with their owners and acquired a large vocabulary of toy names before the beginning of the experiments. Probably for this reason, the process by which these dogs acquired their initial vocabulary of toy labels was only briefly described in the published studies (except in the case of *Chaser*^7^ who received exceptionally intensive professional training). The experimental evidence collected under controlled conditions during these studies^4,7,17^ and in later studies^3^ demonstrate that the GWL dogs required only a few exposures to acquire a novel label. This is in contrast with studies in which dogs required hundreds of repetitions to learn the names of new objects^15,18^.

The extremely small sample size of previous studies undermines the internal and external validity of the findings. The findings cannot be generalized to typical family dogs, as, at the individual level, most typical family dogs do not present behavioural evidence of learning toy names^12–14^. However, it is not known whether they can be generalised to other GWL dogs. Here we aimed at exploring which characteristics may be shared by dogs with a vocabulary of object labels, thereby providing higher validity to past and future findings of studies on GWL dogs as they can be identified as a group with common attributes.

The citizen science approach is popular in ecological research where a rare species is scattered across large geographical areas^19^. In such cases, researchers rely on the observations carried out by amateur naturalists to collect data. As GWL dogs are rare, the search for them and the documentation of their behaviour can most efficiently be done with the help of educated dog owners^20^. Here we harness the power of citizen science to present the first study conducted on a relatively large sample size (N = 41) of dogs that possess a vocabulary of multiple object names. We instructed the owners on how to assess their dogs’ object name knowledge, inquired about the dogs’ life history, the owners’ experience in dog training, and the process by which the dogs learned the names of their toys.

One of our hypotheses is that owners’ skills in dog training or experience with dogs may contribute to the ability of their dogs to learn object names. In this case, we would expect that most owners of GWL dogs are professional trainers or experienced dog owners and that most of them deliberately taught the toy names. Additionally, we aimed to explore whether most GWL dog owners had many dogs in the past and whether their previous dogs or dogs adopted after the GWL dogs, also showed this exceptional ability. Moreover, we expect that households with multiple dogs would likely include more than one GWL dog, assuming that owners have a consistent approach and lifestyle towards their dogs. If the experience gained with each dog would play a role in enabling owners to teach object verbal labels, then we would expect that at least some dogs adopted by the same owners after GWL dogs would become GWL dogs.

Most of the GWL dogs in the literature were Border collies, a breed that has been specifically selected for its trainability^21^. An additional hypothesis for the origin of the ability to learn object labels may be that it is more likely to occur in Border collies, as a result of the breed’s typical characteristics. In this case, we would expect to find GWL dogs only among Border collies. However, the literature also includes reports about two GWL Yorkshire Terriers^3,17^, thus we expect that GWL dogs may also include dogs of several other breeds.

## Results

We used a social media campaign and press articles to locate dogs that, according to their owners’ reports, knew the names of dog toys. The dogs’ knowledge of the toy names was assessed using a two-staged, citizen science model. In the first stage, the owners received instructions on how to test their dog’s knowledge of toy names by themselves (see owner self-conducted test, in the methods section). The second stage was a controlled Vocabulary Assessment Test (VAT), done at the owner’s house with the online presence of the researchers. Owners of dogs that performed significantly above chance in the VAT were asked to complete a questionnaire about the dogs’ life history, their own experience in raising dogs, and the process through which their dog has learned the names of toys. To establish first personal contact with them, we also conducted semi-structured interviews with these owners at the beginning of the online meeting in which we conducted the VAT. Out of the 41 dogs that performed above chance in the VAT, 35 owners completed the questionnaire. As an exploratory analysis for these dogs, we used linear models with AIC-based backwards elimination process, to search for any correlations between variables related to the Demographic Data, Owner Experience in raising and training dogs, the Dogs’ Experience in learning object labels, and the dogs’ Accuracy in the VAT. In addition, we tested for correlation between the number of toys the dogs confirmed to know during the VAT, and the number of toys they were estimated to know approximately two years after the VAT, as well as between the estimated number of toys and the dogs’ Accuracy in the VAT.

### Description of the dog population

Out of the 41 forty-one dogs tested (18 females, 23 males), 23 (56.1%) were Border Collies, 4 (9.8%) Border Collie crosses, 3 (7.3%) Labradors, 2 (4.9%) Pomeranians, 1 (2.4%) Pembroke Welsh Corgi, 1 (2.4%) Miniature Australian Shepherd, 1 (2.4%) Blue heeler x Australian shepherd cross, 1 (2.4%) German Shepherd, 1 (2.4%) Toy Poodle, 1 Golden x Miniature Poodle cross, 1 (2.4%) Shih Tzu, 1 (2.4%) Pekingese, 1 (2.4%) Mongrel (see supplementary spreadsheet). The dogs’ mean age at the time of testing was 3.87 years (SD ± 2.52). The dogs participating in the study were from the USA (n = 17), UK (n = 9), Brazil (n = 5), Canada (n = 3), Norway (n = 2), Hungary (n = 2), Nederland (n = 1), Portugal (n = 1), and Spain (n = 1).

### Vocabulary assessment test (VAT)

All dogs (N = 41) performed significantly above chance in the VAT. Twenty-three dogs (56%) proved to know the names of 20 or more toys. Six (14%) dogs knew 15–19 toy names, five (12%) dogs knew 14–10 toy names, and 7 (17%) dogs knew 5–9 toy names. The individual results of the tests, including the p-value, and chance level for each dog, are available in the supplementary material (supplementary spreadsheet). The maximum number of known toys confirmed for a dog was 86 and the minimum was 5 (mean = 29 toys ± 20 toys). The average Accuracy of the dogs in the VAT (the number of toys the dog successfully retrieved divided by the number of toys upon which it was tested) was 75% (± 15.89%). Since these dogs learn toy names at a very high rate^3,22^, during March and April 2023, we inquired about the current vocabulary size. Thirty-two owners replied to our question. The time that passed from a dog’s initial vocabulary assessment test, until we inquired with the owners about the dog’s current vocabulary size on April 2023 was on average 2 (± 1.13) years. Eight owners reported their dogs knew the names of between 16 and 49 toys, eight owners estimated their dogs’ vocabulary at 50–99 toys, and 16 owners reported that their dogs meanwhile learned over 100 toys (see supplementary spreadsheet).

### Dogs’ life history

Unless specified otherwise, the following results are reported for the 35 dogs for which their owners have completed the questionnaire. Twenty-seven (77%) dogs were obtained from a dog breeder, five dogs (14%) were obtained from a farm, and 3 (9%) dogs were rescued from various locations. All but 2 of the dogs were obtained when they were between 6 and 12 weeks old (average age = 8.13 weeks ± 1.4 weeks). One dog was rescued at the age of 4 weeks, and 1 dog was adopted at the age of 1.5 years. All dogs were given toys to play with when they arrived at the owner’s home.

During the interviews, the owners reported that at the age of adoption, twenty-eight (80%) of the dogs lived in a household with only two caretakers, six dogs (17%) lived in a household with a single caretaker and one dog living in a household with four caretakers (three adults and one teenager). One GWL dog lived with 2 typical (i.e., non-GWL) dogs in the same household and 7 of the GWL dogs lived with one more typical dog in the same household. Out of these, in 4 households, the typical dogs were adopted as puppies when the GWL dogs were adults that already displayed the skill of learning toy names. Three of these 4 typical dogs were of the same breed as the GWL dog but did not show signs of learning toy names. Importantly, there were no 2 GWL dogs in the same family.

Twenty (57%) dogs participated in leisure training activities such as obedience training, agility, dog shows, herding, or scent detection.

### Owners’ experience

Nine (26%) of the owners were first-time dog owners, and for 11 (31%) owners, the current dog was their second dog. For 2 (6%) owners the gifted dog was their third dog and 11 (34%) owners have owned 4 or more dogs in the past. Out of these 25 owners that have owned dogs in the past, 3 (8%) reported that one of their previous dogs knew the names of more than 3 toys. 4 (11%) owners had professional experience as dog trainers. When asked about the source of their knowledge about dog training, 9 (26%) reported using the help of a private dog trainer, 12 (35%) attended dog schools, and 11 (32%) acquired the knowledge from YouTube and books. Three owners (8%) did not report the source of their knowledge.

### The process through which dogs learn toy names

When asked if they intentionally trained their dogs to learn the names of toys, 26 (74%) owners said that initially they did not train the dogs on purpose and started to do so only after noticing that the dogs already knew the names of several toys.

All owners reported that their dogs learned the names of new toys through play interactions. They described the process as follows: (1) presenting the toy in front of the dog, saying the toy’s name; (2) tossing the toy for the dog, sometimes alongside some of the dog’s old toys; (3) asking the dog to retrieve the toy; (4) tugging and pulling on the toy while saying the toy’s name; (5) letting the dog examine and manipulate the toy with their mouth. Owners reported that, when their dogs retrieved the requested toy, they praised and played with them, and some owners also gave them a treat. If the dogs retrieved an incorrect toy, the owners reported correcting the dog and repeating their previous request (e.g., “No, this is not < name of object A > , this is < name of object B > ! Go get < name of object A > !”).

Twenty-eight (80%) owners reported that they play with their dogs with the named toys daily, and 7 (20%) owners reported that they play with the dogs between 2 and 4 times a week. Most owners (n = 31, 88%) reported that the duration of the typical play session is between 1 and 30 min, and 4 (11%) owners reported play sessions that last up to one hour.

When asked how long they believe it takes their dog to learn the name of a new toy, 19 (54%) owners reported 5 min or less, 2 (6%) owners said up to 10 min, 4 (11%) owners reported between 15 and 30 min, 9 (26%) owners replied 1 or 2 days. When asked if there were occasions in which the dogs made frequent mistakes when asked to retrieve a toy, 9 owners reported that the dogs were confused between existing toys if they shared a similar shape, size, and/or material. Twelve owners reported that their dogs confused toys that have similar sounding names or toy names that were composed of several words, some of which were identical at the beginning or the end of the name (e.g., “Butterfly” and “Butterball”, “Starfish” and “Clownfish”). Three owners reported dogs confusing toys that were introduced in temporal proximity to each other (e.g. if the dog received multiple toys during Christmas time). In addition, three owners reported that, if their dogs did not like a specific toy, they had a problem learning the name of that toy.

A summary of characteristics that are shared among the majority (≥ 70%) of GWL dogs’ appears in Table 1.

**Table 1:** characteristics that were found to be shared among 70% or more, of the Gifted Word Learner dogs in the three domains exemined in the study; Demographics, Owner Experience, and Label learning process.

Demographics	Owner experience	Label learning process
Dogs were obtained from breeders	Owners are not professional dog trainers	Dogs learned the object labels by interacting in play sessions with their owners
Dogs lived with their owners from the age of 10 weeks or younger	Owners did not own a GWL dog in the past	Owners did not intentionally teach their dogs the names of dog toys
Dogs were living in a single-dog household	Owners owned at least one dog in the past	Owners estimate that the dogs require less than 30 min to learn the name toy
Dogs live with two adult caretakers		Dogs had a vocabulary of 15 toy names (or more) at the time of the initial Vocabulary Assessment Test
Dogs were neutered or spayed		Dogs were estimated to have reached a vocabulary of over 50 toys (and 50% had an estimated vocabulary of over 100 named toys) approximately two years after the initial test
		Owners play with their dogs with toys daily

### Statistical analyses

The initial linear model that examined the Influence of factors related to Demographic Data on VAT Accuracy included two predictor variables: Sex and AgeAtTest (VATAccuracy ~ Sex + AgeAtTest) (see the methods section for the description of the variables). This model excluded dogs with missing values for AgeAtTest. The predictor AgeAtTest (β = 0.008 ± 0.009, t = 0.953, *p* = 0.347) was not significant. The predictor Sex (β = − 0.091 ± 0.046, t = − 1.986, *p* = 0.054) had emerged as a significant contributor to the model's fit, with males having higher VATAccuracy scores. However, in a subsequent analysis, that included all dogs, the predictor Sex (β = − 0.076 ± 0.491, t = − 1.546, *p* = 0.130) was not significant.

The linear model that examined the Influence of factors related to Owners' Experience, on VAT Accuracy included two predictor variables: NdogsInPast and OwnerTrainingExperience (VATAccuracy ~ NDogsInPast + OwnerTrainingExperience). Both NdogsInPast and OwnerTrainingExperience did not have a significant influence on the model (all *p* ≥ 0.065; See Table S4).

The linear model that examined the Influence of factors related to Dogs’ Experience, on VAT Accuracy included three predictor variables: LeisureTraining, SessionDuration, and LearnDuration (VATAccuracy ~ LeisureTraining + SessionDuration + LearnDuration). None of the predictor variables had a significant influence on VAT Accuracy (all *p* ≥ 0.273, see Table S7).

The Spearman's rank correlation test found a moderate positive correlation between the total number of toys the dogs successfully retrieved during the VAT (ToysVATConfirmed) and the number of toys Estimated by the owners approximately two years later (NToyEstimatesApril23) (r_s_ = 0.45, *p* < 0.05); while there is no correlation between the VAT Accuracy and the number of toys Estimated (NToyEstimatesApril23) (r_s_ = 0.22, *p* = 0.232).

## Discussion

For five years, we searched for dogs that could learn object labels. By utilising a citizen science approach, we located 41 dogs and verified their abilities. During the tests, we found that most of the dogs had already learned over 15 object labels, and their owners reported that they continued to acquire more labels, with many dogs now being able to recognize over 100.

The fact that we failed to find any correlations between factors related to Demographic Data, Owners' Experience, or Dogs’ Experience and Accuracy, may indicate that Accuracy is not a good predictor of object label learning. Accuracy was measured during the first occasion when the dogs were exposed to the setup required for this test. Thus, owners’ excitement, dogs' familiarity with the setup, or their past experiences could have influenced the dog’s performance in the test. We chose to use the dogs’ Accuracy as a dependent variable because it is not influenced by the number of toys a dog has at a given moment. As GWL dogs can rapidly acquire the names of new object labels^3,13,22^, the number of toys the dog has is likely influenced by the owner’s motivation to supply the dog with toys and does not reflect the dogs’ learning abilities.

In the current study, only three out of the 35 owners that completed the questionnaire, reported having a professional background in dog training. In a previous study^13^, we tried to train typical dogs to learn the names of toys by daily playful interactions similar to the ones described by GWL dog owners. Out of the 29 owners who participated in that study, ten had a professional background working with dogs, and yet none of those dogs learned the names of the toys. This supplies additional support to the finding that the owners’ skill in dog training are not the main factor behind the ability of GWL dogs to learn toy names.

Obviously, owners play some role in facilitating GWL dogs’ ability to learn object labels, because the dogs would not be able to learn toy names without owners providing them with toys, and dedicating time to play with them. Our questionnaire is a preliminary exploration into the owners’ experience in handling dogs. Additional studies, conducted under controlled conditions are required to confirm or discard the role of the early experience of the dogs and the skills of the owners in the emergence of the ability to acquire a vocabulary of object names. And yet, the results presented here suggest that the owners’ training skills are likely not the primary driver behind these dogs’ skill. If owners’ skills and commitment were the main factors behind these dogs’ ability to learn object names, we would expect to find multiple GWL dogs in multi-dog households. This point is emphasized by the fact that in three cases, after starting to participate in our study, owners of GWL dogs adopted new puppies. During informal conversations with these owners, we asked them to teach the new puppies the names of toys using the same method that they employed with the older dogs, but according to the owners' reports, the younger dogs did not develop a vocabulary. This suggests that the owners’ experience of interacting with a GWL dog may not be the key requisite for the emergence of the ability to learn in other dogs. Additionally, we would anticipate that experienced owners would report that their previous dogs also knew the names of toys. However, out of the 26 owners who have raised dogs in the past, only three reported that their previous dogs knew the names of a few toys.

Interestingly, 74% of the owners participating in the study reported that they did not intentionally train their dogs to learn the names of toys. Rather, they noticed that their dog had learned toy names, probably during spontaneous playful interactions. Following this realisation, owners began intentionally introducing their dogs to more toys. During interviews, the majority of the owners noted that this spontaneous learning seemed to be effortless (e.g., they reported that the learning process did not seem difficult for the dogs, the dogs learned immediately and performed accurately). Therefore, the owners did not realize how uncommon this ability was until they learned about our research program.

In most of the studies that examine language-related abilities in animals, the subjects are exposed to long and intensive training procedures (see^23^ for a review on Apes for a review on marine mammals^24^, on Gray African parrots^7^, for a study about a GWL dog). Such training procedures may bias the animals to pay specific attention to certain aspects of the trained stimuli, limiting the possibility of reliably demonstrating the animals' innate mental representation of those. Therefore, the finding that GWL dogs, in their natural environment, learn names of toys rapidly and in the absence of intentional training, presents them as an exceptional model for investigating mental representations of objects and their relationship with verbal labels.

According to the owners' estimations, most GWL dogs can learn new toy names in less than 30 min. We consider these reports reliable as they are consistent with previous experimental findings that GWL dogs could learn a new toy name after only four exposures^3^ and can learn two new toy names a day^7,22^. While learning commands and learning object names are distinct skills^14^, it is worth noting that, in contrast to the GWL dogs’ learning speed of object labels, it takes typical family dogs several sessions, each consisting of 20 repetitions, to learn basic commands such as "sit" and "come"^25^.

In our study, we observed that only a few dogs knew less than 10 toy names, and based on the owners' reports, most dogs were capable of learning over 50, or even 100, toy names. While we did not test the dogs' knowledge of hundreds of toys, we considered the owners' reports to be reliable evidence, because their initial reports were confirmed by our vocabulary assessment tests. Previous studies have also used a random sample of a dog's toys to verify the owner's report of the subject's vocabulary^4^.

In a recent personality questionnaire that compared typical Border collies and GWL Border collies, we found that the GWL dogs had higher levels of playfulness^26^. Border collies were selected to work with humans and it has been suggested that high levels of playfulness contribute to improved trainability^21^. Although Border collies’ high playfulness may lead to this breed being over-represented among GWL dogs, we note that even among playful Border collies, object label learning is a rare ability. Attempts to train Border collies to learn the names of toys were not successful^13^, even if, for this study, only dogs that were highly motivated to fetch toys were recruited. In addition, many other dog breeds were selectively bred for working purposes and are characterized by high levels of play motivation (such as German shepherds, Belgian shepherds, Dutch shepherds, etc.). Thus, we suggest that playfulness, by itself, is likely not enough for the development of GWL dogs’ vocabulary. Here we also present the first evidence that, although the ability to learn object labels is most frequently found in Border collies, it also exists in a variety of other breeds.

Our study did not aim to determine the prevalence of GWL dogs in the dog population. Citizen science projects may be limited by their biased population^27^ and our study also suffers from this limitation. Considering the rarity of GWL dogs our sample size is significantly larger than that of all previous studies on this topic. However, we acknowledge the limitations that may stem from a relatively small sample size, also resulting from 6 of the owners not completing the questionnaire. Most of our participants come from English, Portuguese, and Norwegian-speaking countries. This distribution may be a reflection of the media coverage that we have received in these languages. In addition, our recruitment method also limited our outreach to owners with access and proficient users of IT technology and the ability to speak English. To ensure the quality of our research, we established strict screening criteria for owners who wished to participate in our project. By requesting that owners self-test their dogs and submit a video of the test, we ensured that only those who were confident in their dog's ability to learn object names completed the application process.

Citizen science has a significant influence on the research of certain phenomena^27^. Canine citizen science is a relatively new field with the potential to substantially influence canine science^20^. Our study demonstrates that by supplying owners with the necessary guidance and utilizing common technology, canine citizen science can fulfil its potential by allowing researchers to collect reliable scientific data and shed new light on a scarcely documented phenomenon.

Our findings show uniform characteristics that identify GWL dogs as a unique group of dogs. Among these characteristics are the acquisition of a large vocabulary and rapid acquisition of novel labels, characteristics which sharply contrast with the failure of most family dogs to learn object labels^13,14^. This contrast supplies additional support to the hypothesis that GWL dogs vary qualitatively in their object-label learning abilities from the population of typical family dogs^13^. While our findings on GWL dogs should not be generalized to the wider population of typical family dogs, they support previous findings on GWL dogs, increasing their validity and suggesting that what has been found with a specific GWL dog, may be extended to other GWL dogs.

## Methods

### Scientific outreach

Between February 2018 and March 2023, we recruited owners through a social media campaign that included live broadcasts of our experiments (see^22^). These broadcasts and later publications were intensively covered in press articles. While we cannot estimate the exposure our scientific outreach activities achieved, we note that both similarweb.com and semrush.com estimate the online traffic of each of the five biggest media journals that covered our project, at over 300 million readers a month. In addition, popular science articles about the research have been published in at least 12 languages (English, Spanish, German, Russian, Korean, Italian, Dutch, Portuguese, Hungarian, Hebrew, Japanese, and Norwegian).

### Application process

Whenever possible, we asked journalists to include in their coverage of our research a link to an online webpage (http://geniusdogchallenge.com/how-to-apply/) where dog owners who believed their dog knew the names of toys, were encouraged to apply for participation in the research. As data collected during citizen science projects may be of different quality^28^, we used a novel variation of the citizen science model in which participants provide unanalysed data^20^. Our variation of this model included two stages; the first stage, owners' self-conducted tests (see below), served as a screening process during which owners received information on how to independently test their dogs. The second stage, the Vocabulary Assessment Test for the dogs (see below), served for data collection and was conducted with the live guidance and supervision of the researchers. Learning outcomes and participant benefits have been recognized as two factors that contribute to the success of citizen science projects^20^. We addressed these during the second stage, the vocabulary assessment test, where participants received a one-on-one explanation from the researchers about general canine science practices and learned about the existing literature about GWL dogs. In addition, owners of dogs that performed above chance in the vocabulary assessment test were included in an online support community for owners of GWL dogs. As acknowledgment for their contribution, these owners received a certificate, and their dog was featured in the Genius Dog Challenge social media.

### Owners self-conducted test

Owners were asked to complete an application form in English (http://geniusdogchallenge.com/how-to-apply) that provided written instructions for the self-conducted tests and a video guide that explained the rationale behind the testing method (https://www.youtube.com/watch?v=saYjMvoz3S4). The video guide was in English and had the option for generating automatically translated subtitles. To minimize potential inadvertent cues from the owner, the instructions required the owners to place the dog's toys in a different room. Owners were instructed to ask for each of the toys while ensuring that at least three toys were available for the dog to choose from. The application form also included questions regarding the dog’s demographic data (age, breed, sex, and reproduction status), questions about the dog's past, and the owner’s experience in handling dogs. The answers to these questions were not used for data analysis as the questions were repeated in the online questionnaire used for this study (see Questionnaire below). The application form and an example video submitted by one of the owners are available in the supplementary material.

### Vocabulary assessment test (VAT)

After completing the online application and submitting the test video, all owners received an invitation to the online Vocabulary Assessment Test (VAT), during which the researchers collected data to assess the dog's knowledge of the toy names (details of the assessment are provided below). Before the VAT, owners received an email explaining the details of the testing procedure and were asked to send the researchers a list of all the toy names that they believed their dog knew (see supplementary material for the instructions that owners have received).

Before the beginning of the VAT, we summarised the most important results of our research to the owners and asked them some informal questions about their dogs through semi-structured interviews, which allowed owners to become more comfortable with the online testing and interaction with the researchers. During these semi-structured interviews, we encouraged owners to freely describe to us anything that they thought was important about their dog. Among other things, we focused on understanding whether the owner thinks the dog is special (especially in comparison with previously owned dogs). How the dog came about to know the names of toys. The owner’s level of experience in raising and training dogs and whether s/he participates in any other training activities with the dog. We also asked the owners about the presence of other caretakers and animals (including dogs) in the household.

For the test, owners were instructed to use two different recording devices, such as a laptop, tablet, or phone, to connect to an online meeting platform. One device was placed in the room with the dog's toys, while the other was in a room where the owner waited. This setup enabled the experimenter to see the owner, the dog, and the toys throughout the test. The researcher instructed the owner on which toy to ask the dog to retrieve in each trial, following a predetermined random order. The owners asked their dogs to bring the requested toy by saying its name (e.g., "Bring < object name > !"). Typically, the dog went to the toy room, picked a toy, and brought it to the owner in the other room. If the toy was the requested one, owners played with the toy and praised the dog as usual, while some owners also gave their dogs food. In cases where the dogs made a mistake, the owners repeated the request. These repetitions were excluded from the data analysis. If the dog made a second mistake with the same toy, the owner retrieved the toy before moving on to the next trial. Each toy was requested once during the test unless there were mistakes, so the number of trials depended on the number of toys the dog knew. A link to an example of a VAT is available in the supplementary material (video S2).

### Subjects

By March 2023, our citizen science project identified 41 candidate dogs (18 females) that had a significantly higher than-chance performance in the VAT (see results).

### Statistical analysis of the vocabulary assessment tests

To minimize the effects of fatigue we replaced the toys the dog retrieved with either the same ones or new ones after every 1, 3, or 5 trials (as specified in Table 2). This enabled us to test dogs that had many toys, with minimal interruptions. We used individual binomial tests to determine if a dog performed significantly better than chance. The chance level was determined conservatively always taking into account the minimal number of toys that were available for the dog to choose from. This number varied across dogs according to a predetermined scale (see Table 2) based on the number of named toys each dog was reported to know. For example, for a dog that reportedly knew the names of 20 toys, all 20 toys were placed on the floor, and after every five trials the owner placed back the toys the dog had already retrieved. Therefore, in this case, the number of toys from which the dog could choose varied between 20 and 16, and 16 was calculated as the most conservative chance level.

**Table 2:** The Number of toys placed on the floor varied according to the owners’ reports on the number of toys whose dogs knew.

N toy names the dog reportedly know	max N of toys on the floor	N of trials after which the toys were placed back or replaced with new toys	chance level = $$\frac{1}{min n of available toys}$$	N dogs tested
≥ 20	20	5	1/16	27
15–19	15	5	1/11	9
10–14	10	3	1/8	4

Importantly, dogs may have made mistakes during the Vocabulary Assessment Tests, thus not all dogs that reportedly knew over 20 toys, demonstrated this knowledge in the test.

### Statistical analysis of correlation tests

Data analysis was conducted in R statistical environment^29^ using Rstudio^30^. We used Linear Models (lm function) and carried out pairwise comparisons (‘emmeans’ package) for variables that presented significant trends (see the supplementary material for the results of these analyses). In addition, we conducted an AIC-based backwards elimination (drop1 function) to find the most parsimonious models (see supplementary information for the results of this analysis). As the number of toys on which the dogs were tested in the VAT was highly influenced by the owner’s supplying the dog with toys, we used the dogs’ Accuracy level during the test as a dependent variable. Accuracy was calculated by dividing the number of toys the dog successfully retrieved during the test (ToysVATConfirmed), by the number of toys upon which the dog was tested (ToysTestedVAT). We built three separate models to test if Accuracy was affected by factors related to (1) Demographic Data, (2) the Owner’s Experience in handling and training dogs, and (3) factors related to the Dogs’ Experience in learning object labels and training experience.

The linear model for Demographic Data included Sex (female/male) as a factorial variable with two levels, and the dogs' age at the time of the test (Age at Test) as a numerical variable (VATAccuracy ~ Sex + AgeAtTest). As most dogs were adopted from a dog breeder, close to the age of eight weeks and neutered, these three variables (DogOrigin, AgeAdopted and Neutered) could not have been included in the analysis (see supplementary spreadsheets). In addition, as 52% of the dogs in the sample were Border collies and most of the other breeds were represented by only one or two dogs, Breed was also not included in the model. For this initial model, we excluded all dogs that their age at the time of the test was not known (N = 6). In a subsequent analysis, that included only Sex as a predictor of VATAccuracy, we included all 41 dogs that participated in the study.

The model examining the Owner’s Experience on the dogs’ Accuracy included the number of dogs the owner owned in the past (NDogsInPast: 0, 1, 2, ≥ 4; none of the owners reported having 3 dogs) and the level of dog Training Experience the owner’s had (OwnerTrainingExperience: novice; current dog is the first, experienced; owned several dogs in the past, self-educated; owned several dogs in the past and interested in learning dog training methods, dog trainer; has official certification) as factorial variables (VATAccuracy ~ NDogsInPast + OwnerTrainingExperience).

The last model examining the correlation between Accuracy and factors related to the Dogs’ Experience in learning object labels included participation in leisure training activities (LeisureTraining: yes/no) and estimated Learning Duration required to learn a new toy label (LearnDuration: minutes/1–2 days), as a factorial variable with two levels, and the standard duration of a play session in minutes (SessionDuration: 1–10, 10–20, 20–30, 30–60) as a factorial variable with four levels (VATAccuracy ~ LeisureTraining + SessionDuration + LearnDuration). The frequency of play sessions (e.g., the number of times the owner played with the dog in a week) was not included in the model as the majority of owners reported playing with their dogs daily (see supplementary spreadsheets).

To examine whether there was a correlation between the number of toys confirmed during the VAT (ToysVATConfirmed) and the number of toys Estimated two years (± 1.13) after the completion of the VAT (NToyEstimatesApril23), we ran a Spearman's rank correlation test for nonparametric data. Finally, we ran a second Spearman's rank correlation test examining if there is a correlation between the dogs’ Accuracy (VATAccuracy) and the number of toys Estimated (NToyEstimatesApril23).

### Questionnaire

We asked the owners of dogs that performed significantly better than chance in the VAT to complete a questionnaire (see supplementary material). Out of the dogs that performed above chance, 35 owners responded to the questionnaire. The questionnaire covered three topics: (1) information about the dog's life history; (2) the owner's experience with dogs; and (3) the process through which the dog learned the names of toys.

### Informed consent

Informed consent was obtained from the owners of the dogs involved in the study, for their participation in the study and the participation of their dogs.

### Ethical statement

All experiments were performed following relevant guidelines and regulations for the care and use of animals during research. All methods were carried out according to relevant guidelines and regulations for human participants. The Institutional Committee of Eötvös Loránd University has approved the experiments of this study (N. PE/EA/691-5/2019).

### Supplementary Information

Supplementary Information 1.

Supplementary Information 2.

Supplementary Video 1.

Supplementary Video 2.

## Data Availability

Raw data from the Vocabulary Assessment Tests and the owners’ replies to the questionnaire are available in the supplementary material spreadsheet. An additional supplementary document includes an extended method section detailing the recruitment process, the owner questionnaire, and example videos.
