# Supplementary material for: A citizen science model turns anecdotes into evidence by revealing similar characteristics among Gifted Word Learner dogs
Source: Sci Rep. 2023 Dec 14;13:21747. doi: 10.1038/s41598-023-47864-5 (PMC10721640; doi:10.1038/s41598-023-47864-5)
Supplement: Supplementary file 2 — Supplementary Information 2. [file 41598_2023_47864_MOESM2_ESM.docx]

A citizen science model turns anecdotes into evidence by revealing similar characteristics among Gifted Word Learner dogs

Authors: Shany Dror^1,2*^, Ádám Miklósi^1,3, 4^, Andrea Sommese^1^, Claudia Fugazza^1^

*Corresponding author

^1^ Department of Ethology, Eötvös Loránd University, Pázmány P. s 1c, 6th Floor, 1117 Budapest, Hungary

^2^ Doctoral School of Biology, Institute of Biology, ELTE Eötvös Loránd University, Budapest, Hungary

^3^ MTA-ELTE Comparative Ethology Research Group, Budapest, Hungary

^4^ ELTE-ELKH NAP Comparative Ethology Research

# Supplementary material

## Questions asked in the online application form:

(available online at: <https://geniusdogchallenge.com/how-to-apply/>)

- Owner Name
- Email address
- Residents address
- Name of the dog
- Sex: male/female
- Reproductive status: neutered/intact
- Dog’s breed
- Dog’s date of birth
- Pedigree register dog: yes/ no

How many names of objects/toys does your dog know?

Have you purposely trained him/her to learn the names of these objects?

If yes, how long ago did you start training the object names? If not, when did you notice that your Dog knows the names of objects?

Does your dog participate in other training activities such as dog sports or dog shows?

Are there other dogs in the house?

What is your level of experience with dog handling and training?

Is there anything else you would like us to know about your dog?

Instructions for filming the owner self-conduct the test:

To assess your application, we kindly ask you to attach a video demonstrating your dog’s ability. Below are guidelines for filming the video. You can also find an explanatory video on the Genius Dog Challenge YouTube channel (<https://youtu.be/saYjMvoz3S4>)

1. Place all the toys/ objects the dog knows on the floor.
2. Place the camera at a low angle so that both the toys and the entrance to the room are visible. If you are using a mobile phone, please record the video horizontally.
3. Step outside of the room so that the toys are outside of your view and from this position ask the dog to retrieve one toy at a time.
4. Make sure that although we cannot see you in the video, we can hear which toy you are asking for. You can also use a second video camera to record yourself outside of the room but this is not mandatory.
5. Make sure that there are always at least 3 toys on the floor from which your dog can choose. This means that if your dog knows the names of only three toys you need to return the toy, he/ she brought, after each trial before you ask for the next one.

If you have the video of your dog uploaded to a public website (such as YouTube or Facebook) please fill up the link here. If you do not have you’re video uploaded, please upload the video to [WeTransfer](https://wetransfer.com/) and copy the link you receive he.

Video S1- an example of video received from one of the owners during the application process.

<https://youtu.be/NeMZywX_aks>

Supplementary information about the recruitment process:

Between 2020-2023 between 150-200 owners directly contacted us by email and claimed their dogs knew the names of toys. In most cases we referred these owners to our online application form however, if the owners were not proficient users of new technologies, we directly invited them to participate in the Vocabulary Assessment Test (VAT). Overall, through the online application form, we received applications from 35 owners, out of which 21 also submitted videos of their dogs. The rest of the dogs that applied to the project were either directly recruited by the experimenters after the owners posted on social media that their dogs knew the names of object names, or were referred to us by our colleagues or friends.

Supplementary information about the Vocabulary Assessment Test (VAT)

As the number of owners that completed the online application form was relatively low (n=35), all owners that completed the form were invited to participate in the VAT. As GWL dogs are rare and hard to find, we try to make the application process as simple as possible for the owners and always prefer meeting with an owner even if we have little evidence that their dogs knew the names of toys. Fifty-five dogs participated in the VAT. In the manuscript, we report only the results of the dogs that have performed significantly above chance during the test (n=41), as the main focus of the manuscript is to describe the characteristics of GWL dogs, not assess the prevalence of these dogs in the overall population.

We believe it is likely that many owners did not complete the application process because when trying to conduct the Owner Self-conduct test, the dogs did not perform as they expected.

Instructions email owners received before the Vocabulary Assessment Test

About the introduction meeting:

In this meeting, we will get to know each other. We would love to hear about your special dog and explain to you how our project works.

During the meeting, we also aim to complete the first "experiment"/ "test" in which we establish the number of toys your dog currently knows. For this, we will need you to:

1. Send us in advance a list with the names of all the toys your dog knows.

2. Prepare 2 electronic devices with an internet connection (for example your phone and a laptop/ tablet/ someone else's phone). One of these will be placed in the room with you and the other in another room in the house where you will place your dog's toys.

Please try to make sure that your dog is not especially tired before the meeting. You can take your dog out shortly before so s/he can relieve him/ herself but it is better not to exhaust your dog or feed him/her too much before we meet.

We will send you a link to join the meeting. We use an online application called StreamYard. All you will need to do to join the meeting is click on the link we sent you on both of your devices. To prevent echo and other sound problems, it is better if the device you place in the room with the toys is muted and the sound is completely turned off. You might want to use headphones for the meeting, Bluetooth headphones would be the best but if you don't have any headphones we will manage.

Video S2- an example of a Vocabulary Assessment Test, available at: <https://youtu.be/nUp7IdRxWpI>

The questionnaire

1. Name of the dog:‎

2. Sex: female/ male

3. Neutered/ not neutered

4. Dogs birthdate ‎

5. Breed:‎

6. I obtained my dog from a‎

- professional breeder
- farm
- rescued

7. How old was your dog when you adopted him/ her?

8. How many dogs have you owned or trained in the past?‎

- this is my first dog
- 1
- 2
- 3
- 4 or more

9. Do you have another dog in the house at the moment?‎

- No
- 1
- 2
- 3 or more

‎10. Did any of your previous dogs know the names of objects?‎

‎11. Do you participate in any additional activities with your dog?‎ (‎You can choose more than one option)‎

- No
- herding
- agility
- dog shows
- obedience ‎
- dog school

12. How often do you play with your dog the “fetching game” (sending him/ her to ‎retrieve the toys)?‎

‎13. How much experience do you have training dogs?

- This is my first dog.‎
- I have owned several dogs in the past.‎
- I have owned several dogs in the past and self-educated myself about dog training methods (I did not receive any formal education but ‎I have a great interest in this field and have read books about dog training / ‎participated in seminars).‎
- I am a certified dog trainer

‎14. Have you purposely trained your dog to retrieve objects by name?

15. When playing with your dog fetching the toys by name, how long does the play ‎session normally last?‎

16. Have you received professional help or instructions for training your dog?‎

17. How long does it take your dog to learn a new toy?‎

‎18. Can you please describe how you teach your dog the names of toys?‎

19. When you ask your dog to bring a toy and he does not get the correct toy, ‎what do you do?‎

‎20. Does your dog sometimes have a problem learning the name of a new toy? Is ‎there anything about those toys or their names that stands out? ‎

21. How long after receiving your dog did you let him/her play with toys?

Statistical analysis of the correlation tests

**Demographic Data (VATAccuracy ~ Sex + AgeAtTest)**

The AIC-based backwards elimination process led to two models. The full model included Sex and AgeAtTest. The predictor AgeAtTest (df = 1, F = 0.908, p = 0.347) did not significantly influence the model, whereas the predictor Sex (df = 1, F = 3.946, p = 0.054) did significantly influence the model (see table S1). The second model included only the predictors Sex, which still had a significant effect on the model (p = 0.049, see table S2), with males having a higher VATAccuracy values. However, this model excluded dogs with missing values for AgeAtTest (as this is necessary for the comparison between the models). We therefore computed another model which included only the predictor Sex and all the dogs in our sample. In this final model with all dogs, Sex did not have a significant effect (p = 0.130, see table S3).

|  | **Full demographic model** | | | |
| --- | --- | --- | --- | --- |
| *Predictors* | *Estimates* | *CI* | *Statistic* | *p* |
| (Intercept) | 0.771 | 0.674 – 0.868 | 16.097 | **<0.001** |
| Sex _[Females]_ | -0.092 | -0.185 – 0.002 | -1.986 | 0.054 |
| AgeAtTest | 0.009 | -0.010 – 0.027 | 0.953 | 0.347 |
| Observations | 35 | | | |
| R^2^ / R^2^ adjusted | 0.120 / 0.073 | | | |

Tadel S1: the full demographic model. Dogs with missing values are excluded.

|  | **second demographic model** | | | |
| --- | --- | --- | --- | --- |
| *Predictors* | *Estimates* | *CI* | *Statistic* | *p* |
| (Intercept) | 0.806 | 0.743 – 0.868 | 26.137 | **<0.001** |
| Sex _[Females]_ | -0.094 | -0.187 – -0.001 | -2.038 | **0.049** |
| Observations | 35 | | | |
| R^2^ / R^2^ adjusted | 0.099 / 0.075 | | | |

Tadel S2: the second demographic model. Dogs with missing values are excluded.

|  | **All dogs demographic model data** | | | |
| --- | --- | --- | --- | --- |
| *Predictors* | *Estimates* | *CI* | *Statistic* | *p* |
| (Intercept) | 0.788 | 0.722 – 0.854 | 24.191 | **<0.001** |
| Sex _[Females]_ | -0.076 | -0.176 – 0.023 | -1.546 | 0.130 |
| Observations | 41 | | | |
| R^2^ / R^2^ adjusted | 0.058 / 0.034 | | | |

Tadel S3: the final demographic model Including only the predictor Sex and data from all the dogs that participated in the study.

**Owner’s Experience** **(VATAccuracy ~ NDogsInPast + OwnerTrainingExperience)**

The model included two predictor variables: NDogsInPast and OwnerTrainingExperience, both did not significantly improve the model (NDogsInPast; df = 3, F = 2.387, p = 0.091, OwnerTrainingExperience; df = 3, F = 1.425, p = 0.257, see table S4). As the predictor NDogsInPast presented a trend for the comparison between zero dogs and four or more dogs owned in the past (table S4), we conducted an additional analysis, using the emmeans function for pairwise comparisons, and the tukey method for adjustments of p values for multiple comparisons. In this post hock test we did not find any significant differences between the VATAccuracy scores of dogs owned by owners that owned different numbers of dogs in the past (table S5). The predictor OwnerTrainingExperience, presented a trend for the comparison between experienced owners and owners that were professional dog trainer (table S4). Here again we conducted an additional analysis using the emmeans function for pairwise comparisons, and the tukey method for adjustments of p values for multiple comparisons and did not find any significant differences (table S6).

|  | **Owners' Experience - first model** | | | |
| --- | --- | --- | --- | --- |
| *Predictors* | *Estimates* | *CI* | *Statistic* | *p* |
| (Intercept) | 0.655 | 0.495 – 0.815 | 8.415 | **<0.001** |
| NDogsInPast _[0]_ | 0.148 | -0.012 – 0.308 | 1.900 | 0.068 |
| NDogsInPast _[1]_ | 0.004 | -0.147 – 0.155 | 0.055 | 0.956 |
| NDogsInPast _[2]_ | 0.162 | -0.050 – 0.373 | 1.570 | 0.128 |
| OwnerTrainingExperience_[Experienced]_ | 0.207 | -0.014 – 0.428 | 1.926 | 0.065 |
| OwnerTrainingExperience_[Novice]_ | 0.045 | -0.139 – 0.230 | 0.505 | 0.618 |
| OwnerTrainingExperience_[SelfEducated]_ | 0.025 | -0.134 – 0.183 | 0.317 | 0.753 |
| Observations | 34 | | | |
| R^2^ / R^2^ adjusted | 0.271 / 0.108 | | | |

Table S4: the Owner Experience model. The numbers in subscript square brackets present the number of dogs owned in the past, with the reference category being an owner owning four or more dogs. For the OwnerTrainingExperience, the reference category was the owner receiving official education as a dog trainer.

|  | **pairwise comparisons for NDogsInPast** | | | |
| --- | --- | --- | --- | --- |
| contrast | estimate | *SE* | t.ratio | *p* |
| ≥4 - 0 | -0.148 | 0.078 | -1.900 | 0.252 |
| ≥4 - 1 | -0.004 | 0.074 | -0.055 | 0.999 |
| ≥4 - 2 | -0.162 | 0.103 | -1.570 | 0.412 |
| 0 - 1 | 0.144 | 0.068 | 2.123 | 0.171 |
| 0 - 2 | -0.014 | 0.104 | -0.131 | 0.999 |
| 1 - 2 | -0.158 | 0.099 | -1.584 | 0.404 |
| df | 27 | | | |

Tabel S5: post hock tests of the pairwise comparisons examining differences in VATAccuracy in owners with varying levels of experience in owning dogs (NDogsInPast). The numbers under the contrast column present the comparisons between the four possible factors: ≥4; owning four or more dogs, 0; the current dog is the first dog, 1; one dog owned in the past, 2; owning two dogs in the past (non of the owners participating in this study owned three dogs in the past).

|  | **pairwise comparisons for** **OwnerTrainingExperience** | | | |
| --- | --- | --- | --- | --- |
| contrast | estimate | *SE* | t.ratio | *p* |
| DogTrainer – ExperiencedOwner | -0.207 | 0.108 | -1.926 | 0.241 |
| DogTrainer - NoviceOwner | -0.045 | 0.090 | -0.505 | 0.957 |
| DogTrainer - SelfEducatedOwner | -0.024 | 0.077 | -0.317 | 0.989 |
| Experienced - NoviceOwner | 0.162 | 0.109 | 1.486 | 0.459 |
| Experienced - SelfEducatedOwner | 0.183 | 0.097 | 1.890 | 0.256 |
| Novice - SelfEducatedOwner | 0.021 | 0.066 | 0.315 | 0.989 |
| df | 27 | | | |

Tabel S6: post hock tests of the pairwise comparisons examining differences in VATAccuracy in owners with varying levels of experience training dogs (OwnerTrainingExperience).

**Dogs’ Experience** **(VATAccuracy ~ LeisureTraining + SessionDuration + LearnDuration)**

The AIC-based backwards elimination process led to three models. The full model included three predictor variables: LeisureTraining, SessionDuration, and LearnDuration. All three predictor variables did not influence the model (LeisureTraining; df = 1, F = 0.417, p = 0.524, SessionDuration; df = 3, F = 0.635, p = 0.599, LearnDuration; df = 1, F = 0.484, p = 0.492; see table S76). The second model included LeisureTraining and LearnDuration. These predictor variables did not influence the model (LeisureTraining; df = 1, F = 0.759, p = 0.390, LearnDuration; df = 1, F = 0.197, p = 0.660). The third model included only LeisureTraining, which did not significantly influence the model (LearnDuration; df = 1, F = 0.619, p = 0.437).

|  | **Dogs’ Experience - first model** | | | |
| --- | --- | --- | --- | --- |
| *Predictors* | *Estimates* | *CI* | *Statistic* | *p* |
| (Intercept) | 0.782 | 0.658 – 0.906 | 12.977 | **<0.001** |
| LeisureTraining _[yes]_ | -0.043 | -0.179 – 0.094 | -0.646 | 0.524 |
| SessionDuration_10-20_ | -0.016 | -0.183 – 0.152 | -0.192 | 0.849 |
| SessionDuration_20-30_ | -0.017 | -0.166 – 0.131 | -0.237 | 0.815 |
| SessionDuration_30-60_ | 0.108 | -0.090 – 0.305 | 1.119 | 0.273 |
| LearnDuration _[1-2days]_ | -0.050 | -0.198 – 0.098 | -0.696 | 0.492 |
| Observations | 33 | | | |
| R^2^ / R^2^ adjusted | 0.088 / -0.081 | | | |

Tabel S7: the first Dogs’ Experience model. The reference category for LeisureTraining was not Participating in any leisure activities. The reference category for session duration was standard session duration of 1-10 minutes. The reference category for LearnDuration was a learning duration of minutes.
